# Engineered exosomes restore miR-508-5p expression in uterine corpus endometrial carcinoma and reduce tumor progression and metastasis by targeting DLL3

**DOI:** 10.3389/fonc.2025.1532564

**Published:** 2025-02-24

**Authors:** Yue-Ying Li, Hui Liu, Jia-Lu Feng, Wen-Yan Tian, Juan Du, Li-Ping Zhang

**Affiliations:** ^1^ Department of Gynecology and Obstetrics, Tianjin Medical University General Hospital, Tianjin, China; ^2^ Tianjin Key Laboratory of Female Reproductive Health and Eugenic, Tianjin Medical University General Hospital, Tianjin, China; ^3^ Department of Neonatology, TaiHe Hospital, Hubei University of Medicine, Shiyan, Hubei, China; ^4^ Department of Medicine, Wuhan University of Science and Technology, Wuhan, Hubei, China; ^5^ Department of gynecology, Wuhan Children’s Hospital (Wuhan Maternal and Child Healthcare Hospital), Tongji Medical College, Huazhong University of Science & Technology, Wuhan, Hubei, China

**Keywords:** endometrial cancer, exosomes, miRNA, DLL3, tumor progression

## Abstract

**Introduction:**

Endometrial cancer (EC) is a growing global health concern. Understanding the molecular mechanisms driving EC is crucial for developing effective diagnostic and therapeutic strategies. This study investigates the roles of DLL3 and miR-508-5p in EC progression and explores a therapeutic approach using engineered exosomes to modulate their expression.

**Methods:**

TCGA data were analyzed, *in vitro* and *in vivo* experiments were performed to assess DLL3 and miR-508-5p function, and bioinformatics was used to confirm their interaction. Mesenchymal stem cells (MSCs) were engineered to produce miR-508-5p-overexpressing exosomes, and their therapeutic effects were tested in mouse models.

**Results:**

Elevated DLL3 and downregulated miR-508-5p were observed in EC and correlated with poor outcomes. miR-508-5p directly targets DLL3. Engineered exosomes restored miR-508-5p, inhibited DLL3, and reduced tumor growth and metastasis in mouse models.

**Discussion:**

The findings highlight the roles of DLL3 and miR-508-5p in EC. Targeting the miR-508-5p/DLL3 axis via exosome-mediated delivery represents a promising therapeutic strategy for EC.

## 1 Introduction

Endometrial cancer (EC) ranks as the sixth most common cancer in women worldwide (1), with a lifetime risk of approximately 3% for women. Over the past 30 years, its overall incidence has increased by 132% (2). The external risk factors for uterine corpus endometrial (UCEC) are primarily obesity, metabolic, and reproductive factors, while the intrinsic risk factors revolve around genetics and epigenetics (3). Consequently, with the rising rates of obesity and aging in modern society, the incidence of UCEC is also on the increase.

Historically, UCEC has been categorized into two broad types: estrogen-dependent (Type I) and estrogen-independent (Type II) cancers. Type I, consisting of low and intermediate-grade endometrioid ECs (EECs), is the most common (70%) and is associated with hormone receptor positivity (4, 5). Generally, Type I has a favorable prognosis. Type II includes high-grade EEC and non-endometrioid subtypes such as serous (SEC), clear cell (CCC), carcinosarcoma (CS), and undifferentiated EC. These tumors are not associated with estrogen, obesity, and have a poor prognosis (5). UCEC is generally considered a cancer with a favorable outcome. However, with the rising incidence, it has been recognized that not all ECs have a favorable prognosis (6). This applies not only to Type II tumors but also to some Type I tumors, which can exhibit unexpectedly aggressive behavior.

Delta-like ligand 3 (DLL3), a member of the Notch ligand family, is a transmembrane protein anchored to the cell surface. The human DLL3 protein, composed of 619 amino acids, is characterized by a DSL domain, six EGF-like repeat sequences, and a transmembrane domain. DLL3 can bind to Notch receptors (Notch 1-4) to activate the Notch pathway, a highly conserved cell signaling pathway associated with malignant transformation, cell proliferation, cell cycle arrest, apoptosis, epithelial-mesenchymal transition (EMT), and inhibition of neuroendocrine differentiation (7). DLL3 is broadly expressed in neuroendocrine carcinomas (NECs), including lung NEC, gastrointestinal pancreatic, bladder, prostate, and cervical NECs (8). Numerous studies have consistently shown that DLL3 is an attractive target for cancer immunotherapy. The DLL3-targeting antibody-drug conjugate (ADC) rovalpituzumab tesirine (Rova-T, SC16LD6.5) has demonstrated durable *in vivo* tumor regression across a variety of patient-derived xenograft models (9).

MicroRNAs (miRNAs) are a class of endogenously encoded, approximately 22 nucleotide long non-coding single-stranded RNA molecules that participate in the post-transcriptional regulation of gene expression in both animals and plants (10). They are highly conserved across species in regulating gene expression, primarily through the translation suppression or degradation of messenger RNA (mRNA). miRNAs regulate target mRNAs by destabilizing them or inhibiting their translation (11). Mature miRNAs are actively involved in the proliferation, migration, apoptosis, metabolism, and other cellular responses of every subtype of human cancer (12).

MicroRNA-508-5p (miR-508-5p) has been shown to regulate and reverse multidrug resistance in gastric cancer, with its absence reducing drug sensitivity (13). MiR-508-5p serves as a prognostic marker inhibiting the proliferation and migration of glioma cells (14, 15), indicating its role in regulating tumor onset and progression.

The roles of miR-508-5p, DLL3, and their involvement in cancer development and progression are closely linked. However, current knowledge on DLL3 and UCEC is limited to a single study analyzing the cancer genome atlas (TCGA) database, which suggests that DLL3 overexpression and advanced tumor stage, grade, and lymph node metastasis are independent prognostic predictors for EC. DLL3 expression could be a potential new biomarker for early diagnosis and an independent predictor of poor survival in UCEC patients (16). Research on miR-508-5p and UCEC is also sparse.

Exosomes, with diameters ranging from 30 to 150 nm, are extracellular vesicles originating from endosomes (17). These vesicles carry proteins, lipids, polysaccharides, and nucleic acids from their parent cells (18), which have been shown to regulate intercellular communication (19). Compared to other delivery vehicles, exosomes overcome natural barriers, exhibit minimal toxicity, possess superior biocompatibility, and can efficiently and precisely deliver their cargo to tumor sites (20). Notably, numerous studies have identified mesenchymal stem cell (MSC)-derived exosomes as powerful candidates for anti-tumor therapy. For instance, adipose-derived mesenchymal stem cells (ADMSCs) transfected with miR-122 can effectively transfer miR-122 to hepatocellular carcinoma cells, sensitizing these cells to 5-FU and sorafenib by downregulating miR-122 target genes (21). Similarly, exosomes from miR-199-modified ADMSC can inhibit the growth of hepatocellular carcinoma by suppressing the mechanistic Target of Rapamycin (mTOR) signaling pathway (22). These findings highlight the significant potential of MSC-derived exosome therapy in cancer treatment.

In this study, the expression data for 33 cancers and their corresponding adjacent tissues were downloaded from the TCGA database, revealing abnormal DLL3 expression levels in various tumors, UCEC, where DLL3 expression was significantly upregulated and correlated with shorter overall survival (OS) in patients. Further experimental validation demonstrated that reducing DLL3 expression in tumor cells significantly inhibited their proliferation, migration, and invasion capabilities. A detailed transcriptome analysis of UCEC showed significant downregulation of miR-508-5p in tumor tissues, with bioinformatics predictions identifying a potential binding site for miR-508-5p on the 3’UTR of DLL3. Dual-luciferase reporter assays confirmed the direct binding between miR-508-5p and the DLL3 3’-UTR, elucidating the regulatory role of the miR-508-5p/DLL3 axis in the metastatic process of UCEC cells. Engineered mesenchymal stem cells overexpressing miR-508-5p were developed, and exosomes were collected using iodixanol gradient density ultracentrifugation to restore miR-508-5p expression in UCEC cells. The exosomes were efficiently taken up by UCEC cells, restoring miR-508-5p expression and significantly inhibiting DLL3 expression, leading to the suppression of UCEC cell proliferation and metastasis. This resulted in notable tumor regression and reduction of metastatic foci in cell-derived xenograft (CDX) and tail vein lung metastasis mouse models. In summary, our research provides robust experimental evidence for understanding the regulatory mechanisms of the miR-508-5p/DLL3 axis in the development of EC and introduces a novel therapeutic method based on engineered exosome delivery to interfere with their expression.

## 2 Materials and methods

### 2.1 Cell culture

The human endometrial carcinoma cell lines, HEC-1-A and AN3CA, were propagated in McCoy’s 5A (Gibco, USA) and Minimum Essential Medium (MEM, Gibco, USA), respectively, as obtained from the Cell Bank of the Chinese Academy of Sciences. The media were supplemented with 10% fetal bovine serum (FBS, Gibco, USA) and 1% penicillin-streptomycin. Human endometrial epithelial cells (HEEC) were also sourced from the Cell Bank of the Chinese Academy of Sciences and were cultured in DMEM/F12 medium (Gibco, USA) with 10% FBS and 1% penicillin-streptomycin. Human mesenchymal stem cells (hMSCs), purchased from Wuhan Punuosai Life Technology (Wuhan, China), were cultured in serum-free medium specifically designed for hMSCs by Wuhan Punuosai Life Technology. All cells were cultivated in a controlled environment at 37°C with a 5% CO_2_ atmosphere. When reaching 80-90% confluence, subculturing was performed using 0.25% trypsin-EDTA, ensuring cell integrity and experimental reproducibility.

### 2.2 Plasmid construction

The full 3’ untranslated region (3’UTR) of DLL3 was subsequently cloned into the pmirGLO vector (Promega, USA) for post-transcriptional regulation analysis. MiR-508-5p mimics and inhibitors, along with the aforementioned plasmids, were synthesized by Qingke Biotech Co., Ltd. (China). Verification of all constructs was achieved through Sanger sequencing.

### 2.3 CCK-8 assay

The Cell Counting Kit-8 (CCK-8, Vazyme, China) assay was utilized to evaluate cell viability and proliferation post-transfection. Cells were plated in 96-well plates at densities tailored to each cell line’s growth characteristics and incubated for 24 hours to ensure adequate attachment. Following this period, CCK-8 solution was carefully added to each well, and the plates were incubated for a duration of 1-4 hours at 37°C in a 5% CO2 atmosphere. The optical density at 450 nm was determined using a microplate reader (BioTek Instruments, USA), providing a quantitative measure of cell viability. To ensure the reliability of the results, the assay was conducted in triplicate and averaged for statistical analysis.

### 2.4 Colony formation assay

For the colony formation assay, 500 cells were plated at low density in 6-well plates and cultured for 14 days, with the medium refreshed every 3 days. Colonies were fixed with methanol and stained with 0.1% crystal violet (Beyotime Biotechnology, China). The number of colonies, defined as a group of more than 50 cells, was counted manually using an Olympus light microscope (Olympus Corporation, Japan).

### 2.5 Wound scratch assay

A confluent cell monolayer in a 6-well plate was scratched using a sterile pipette tip to create a wound. Detached cells were removed by washing, and the migration of cells into the wound area was documented at 0 and 48 hours using an Olympus phase-contrast microscope (Olympus Corporation, Japan). The wound closure percentage was calculated as (1 - 48h area/0h area) x 100%, then normalized to negative controls (NC, set as 1). Relative Closure = (Experimental group Closure %)/(NC Closure %).

### 2.6 Transwell migration and invasion assays

Cell migration capabilities were assessed using Corning Transwell chambers (8.0 µm pore size). Cells were seeded into the upper chamber in serum-free media, whereas the lower chamber was filled with media supplemented with 10% FBS to serve as a chemoattractant. After a period of 24-48 hours, cells that had migrated through the membrane were fixed with 4% paraformaldehyde, stained with 0.1% crystal violet, and air-dried. For quantification, four randomly selected fields per membrane were imaged under an inverted microscope. Cells were manually counted and the averaged cell count per field was normalized to the negative control (NC) group (set as 1) to determine relative migration capacity.

### 2.7 Western blot

Cells were lysed and protein concentrations were determined using a BCA protein assay kit (Thermo Fisher Scientific, USA). Equal amounts of protein were separated on SDS-PAGE gels and transferred to polyvinylidene fluoride (PVDF) membranes (Merck Millipore, Germany). Membranes were blocked and then incubated with a primary antibody against DLL3 (Abclonal, China, A18108), CD63 (Abclonal, China, A19023), CD81 (Abclonal, China, A4863), TSG101 (Abclonal, China, A1692), Calnexin (Abclonal, China, A4846). Following primary incubation, membranes were treated with HRP-conjugated secondary antibody (Abclonal, China, AS014). Protein bands were visualized using enhanced chemiluminescence (ECL) reagents (Meilunbio, China) and quantified with image analysis software.

### 2.8 Quantitative real-time PCR

Total RNA was extracted using the FastPure Cell/Tissue Total RNA Isolation Kit V2 (Vazyme, China), and miRNA was specifically extracted with the miRNeasy Kits (Qiagen, Germany), following the protocols provided by the manufacturers. Reverse transcription for mRNA was performed using the HiScript III 1st Strand cDNA Synthesis Kit (+gDNA wiper) (Vazyme, China), while miRNA reverse transcription utilized the miRNA 1st Strand cDNA Synthesis Kit (by stem-loop) (Vazyme, China).

qRT-PCR analyses were executed on a Bio-Rad CFX system using SYBR Green Master Mix (Vazyme, China). The primers used targeted DLL3, GAPDH, U6, and miR-508-5p, respectively. DLL3 primer F: CGTAGATTGGAATCGCCCTGAAG, R: CGTAGATGGAAGGAGCAGATATGAC, GAPDH primer F: ACAACTTTGGTATCGTGGAAGG, R:GCCATCACGCCACAGTTTC, U6(F: CTCGCTTCGGCAGCACA, R: AACGCTTCACGAATTTGCGT), miR-508-5p F: TACTCCAGAGGGCGTCACTCATG.

### 2.9 Bioinformatics assay

Gene expression data, corresponding survival data, and clinical information for patients were downloaded from the UCSC Xena database (TCGA-UCEC cohort) (https://xenabrowser.net/). For survival analysis, patients were stratified into high and low DLL3 expression groups based on the median expression value of DLL3. Kaplan-Meier survival curves were generated and compared using the log-rank test, implemented in the ‘survival’ R package (23). Univariate Cox proportional hazards regression analysis was also conducted using the ‘survival’ package to assess the prognostic significance of DLL3 expression (24). Differential gene expression analysis between the high and low DLL3 expression groups was performed using the ‘edgeR’ package in R. Genes with an adjusted p-value (FDR) &lt; 0.05 and |log2FoldChange| > 2 were considered differentially expressed. A volcano plot was generated using the ‘ggplot2’ package (25) to visualize the differentially expressed genes. Gene Ontology (GO) and Kyoto Encyclopedia of Genes and Genomes (KEGG) pathway enrichment analyses were performed using the ‘clusterProfiler’ package to identify significantly enriched biological processes and pathways associated with the differentially expressed genes (26). Gene Set Enrichment Analysis (GSEA) was performed using the ‘clusterProfiler’ package and the ‘ReactomePA’ package to further investigate the functional implications of DLL3 expression in UCEC (26, 27). The R software version used for all of the above analyses is 4.4.1.

### 2.10 Immunohistochemistry

Paraffin-embedded tissue sections underwent deparaffinization and rehydration, followed by antigen retrieval in a citrate buffer solution. The sections were then blocked to prevent non-specific binding and incubated with a primary antibody against DLL3 (Abclonal, China, A18108) overnight at 4°C. This step was succeeded by the application of HRP-conjugated secondary antibodies (Abclonal, China, AS014). For visualization, DAB (3,3’-Diaminobenzidine) was utilized as the chromogen, with sections subsequently counterstained with hematoxylin to highlight the nuclei. The prepared slides were examined under a light microscope. Quantitative assessment of staining intensity was meticulously performed using specialized image analysis software ImageJ, ensuring precise and objective evaluation of the expression levels of DLL3 in the tissue samples.

### 2.11 Dual-luciferase reporter assay

The 3’UTR regions of DLL3 were cloned into the pmirGLO vector, alongside control vectors, and co-transfected into target cells with miR-508-5p mimics or control sequences. Luciferase assays were conducted using the Dual-Luciferase Reporter Assay System (Beyotime, China), following manufacturer’s protocols. Firefly luciferase activity was measured and normalized against Renilla luciferase to assess relative luciferase expression. Measurements were performed using a microplate luminometer (Bio-Rad, USA).

### 2.12 RNA immunoprecipitation experiment

The RIP experiment was carried out using an RIP Kit (Thermo Fisher Scientific, USA) following the provided protocol. Briefly, cells were lysed, and the lysates were incubated with magnetic beads conjugated to antibodies against Argonaute 2 (Ago2, Abclonal, China, A19709) or non-specific IgG (AC005, Abclonal, China) as a control. After incubation, RNA bound to the beads was isolated. The expression of miR-508-5p and DLL3 mRNA in the immunoprecipitated complexes was detected via qRT-PCR.

### 2.13 Clinical tissue sample collection

From 2023 to 2024, clinical samples were collected from Wuhan Children’s Hospital. The collection of clinicopathological samples was approved by the Medical Ethics Committee of Wuhan Children’s Hospital in accordance with the Declaration of Helsinki (approval number: 2024R045), with approval granted from May 6, 2024, to May 5, 2025. Immediately after surgery, the samples were submerged in liquid nitrogen, and subsequently, Wuhan Servicebio Technology Co., Ltd., was entrusted with the paraffin embedding of the samples.

### 2.14 Preparation of engineered exosomes

To construct engineered MSCs, lentivirus overexpressing miR-508-5p was used to establish stable miR-508-5p overexpressing MSCs. The lentivirus was purchased from GeneChem (Shanghai, China). Briefly, MSCs were infected with the lentivirus, followed by puromycin selection 72 hours post-infection. Single MSC clones were picked, expanded, cultured, and confirmed for miR-508-5p expression.

The supernatant from the cultured engineered MSCs was collected into centrifuge tubes. The first centrifugation was performed at 500g for 10 minutes at 4°C, and the supernatant was carefully transferred to new centrifuge tubes. A second centrifugation was done at 2,000g for 20 minutes at 4°C, followed by supernatant collection. The third centrifugation was at 12,000g for 20 minutes at 4°C, with the supernatant collected and filtered using a 0.22μm filter.

For ultracentrifugation, a Beckman ultracentrifuge with an SW 32 Ti rotor was used at 120,000g for 90 minutes at 4°C. After centrifugation, the pellet was resuspended in PBS, aliquoted, and stored at -80°C.

### 2.15 Transmission electron microscopy assay

Exosome samples were negatively stained for TEM imaging. The exosome samples were placed on 200-mesh copper grids. They were then stained with 1% phosphotungstic acid (Aladdin, China) for 1 minute. Subsequently, the samples were washed twice with PBS, each for 1 minute, and the grids were dried before visualization using a Hitachi HA7100 transmission electron microscope, operated at 80 kV.

### 2.16 Nano-flow cytometry assay

Purified engineered exosomes overexpressing miR-508-5p were analyzed for particle size and concentration using a flow nanoanalyzer (nanoFCM, China). S23M-SEV (nanoFCM, China) was utilized as the particle size standard, while the Quality Control Nanospheres Series (nanoFCM, China) served as the concentration standard. The data were processed using NF Profession 1.16 software.

### 2.17 Animal model

Nude mice (6-8 weeks old, female) were obtained from the Laboratory Animal Center at Wuhan University of Science and Technology, Wuhan, China. For the subcutaneous xenograft model, 5 × 10^6^ UCEC cells were resuspended in 100 µL of medium and injected subcutaneously into each mouse. In the metastasis model, 1 × 10^6^ UCEC cells were injected via the tail vein using a murine tail vein injector in a volume of 100 µL. The study was conducted in accordance with the guidelines of the Wuhan University of Science and Technology’s Animal Care and Use Committee, with ethical approval (2024116). Tumor growth was monitored by caliper measurements, and mice were euthanized when tumors reached 1500 mm³ in compliance with humane endpoints. Tumors were subsequently collected for further examination. In the metastasis model, euthanasia was performed 45 days post-injection, followed by isolation of lung tissue for HE staining.

### 2.18 Statistical analysis

Data were presented as mean ± standard deviation (SD) from at least three independent experiments. Statistical significance between groups was evaluated using Student’s t-test or one-way ANOVA, followed by *post-hoc* tests when applicable. A p-value < 0.05 was considered statistically significant. All analyses were performed using SPSS or GraphPad Prism software.

## 3 Result

### 3.1 Aberrant expression of dll3 in various cancers and its significant association with survival rates in endometrial cancer patients

**Figure 1:**
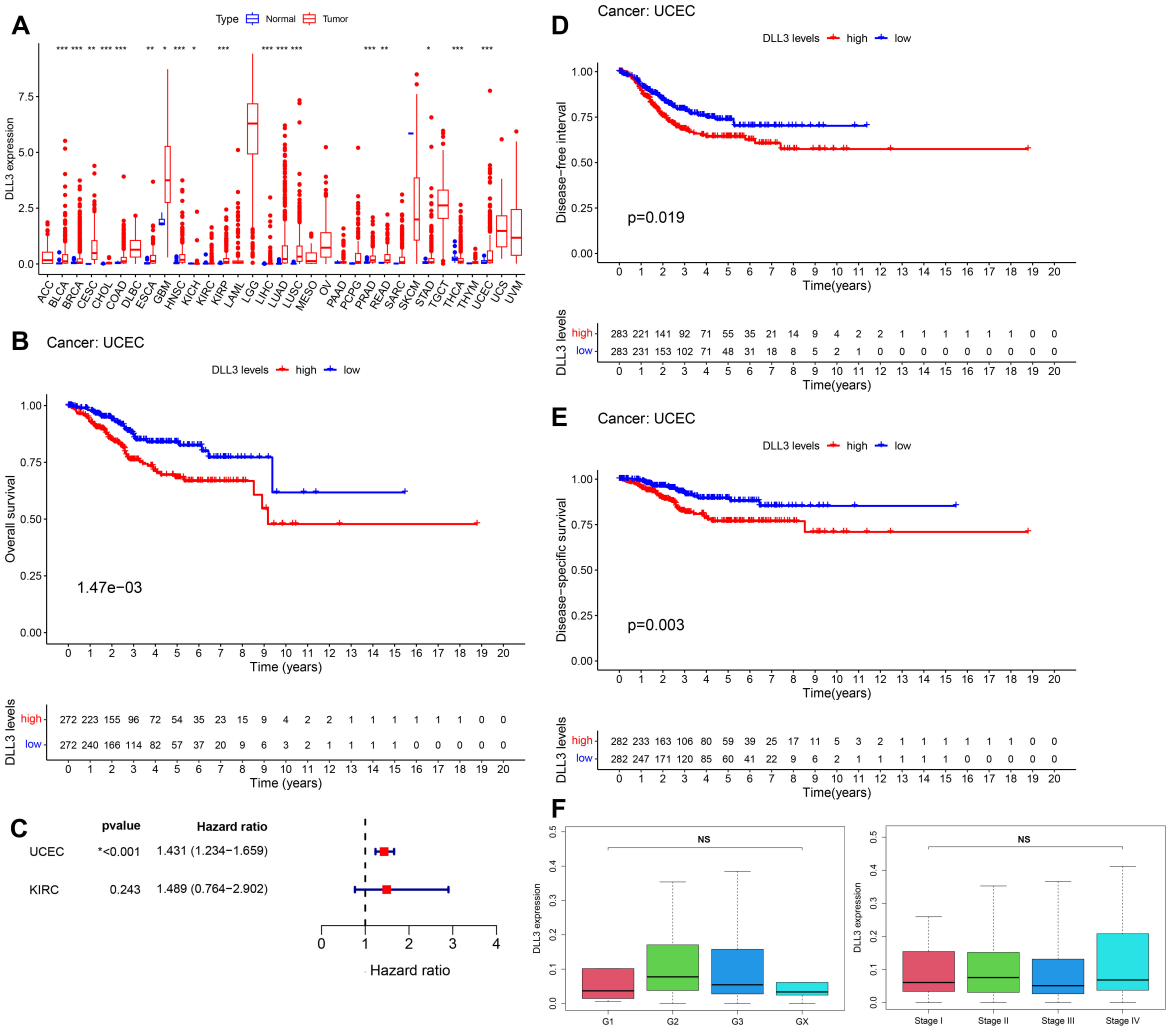
Aberrant expression of DLL3 in various cancers and its significant association with survival rates in endometrial cancer patients. **(A)** Expression of DLL3 in 33 types of cancers, data derived from the TCGA database. **(B)** Kaplan-Meier curve illustrating the correlation between DLL3 expression and OS in UCEC, with statistical analysis by Log-rank test. **(C)** Survival analysis using Cox proportional hazards model to assess the significance of DLL3 expression in UCEC and KIRC. **(D)** DFI curve for DLL3 expression in UCEC. **(E)** DSS curve for DLL3 expression in UCEC. **(F)** Analysis of the correlation between DLL3 expression and patient grade and stage. The results presented were Mean ± SD. *p < 0.05, **p < 0.01, ***p < 0.001, NS > 0.05.

In an in-depth analysis of the expression profiles for 33 cancer types within the TCGA database, a lack of adjacent non-tumor tissue data precluded statistical analysis for 10 types of cancer. Among the remaining 22 cancer types, DLL3 expression was found to be downregulated in thyroid cancer (THCA) but significantly increased in several other cancers compared to adjacent non-tumor tissues (**Figure 1A**). Further survival data analysis of cancer types with significant changes in expression revealed that the expression level of DLL3 was significantly associated with overall survival (OS) in patients with renal cell carcinoma (KIRC) and UCEC, as indicated by Log-rank tests. However, results from the Cox proportional hazards model showed that high DLL3 expression was significantly associated with poor survival prognosis only in UCEC patients (**Figures 1B, C**). Additionally, for UCEC patients, those with high DLL3 expression had significantly worse disease-free interval (DFI) and disease-specific survival (DSS) (**Figures 1D, E**). Further analysis revealed no significant correlation between DLL3 expression and the patients’ grade or stage (**Figure 1F**), suggesting that DLL3 expression is independent of the clinical staging and has potential as an independent prognostic factor for cancer.

### 3.2 Transcriptomic and functional enrichment analysis uncovers the role of DLL3 in UCEC pathogenesis

**Figure 2:**
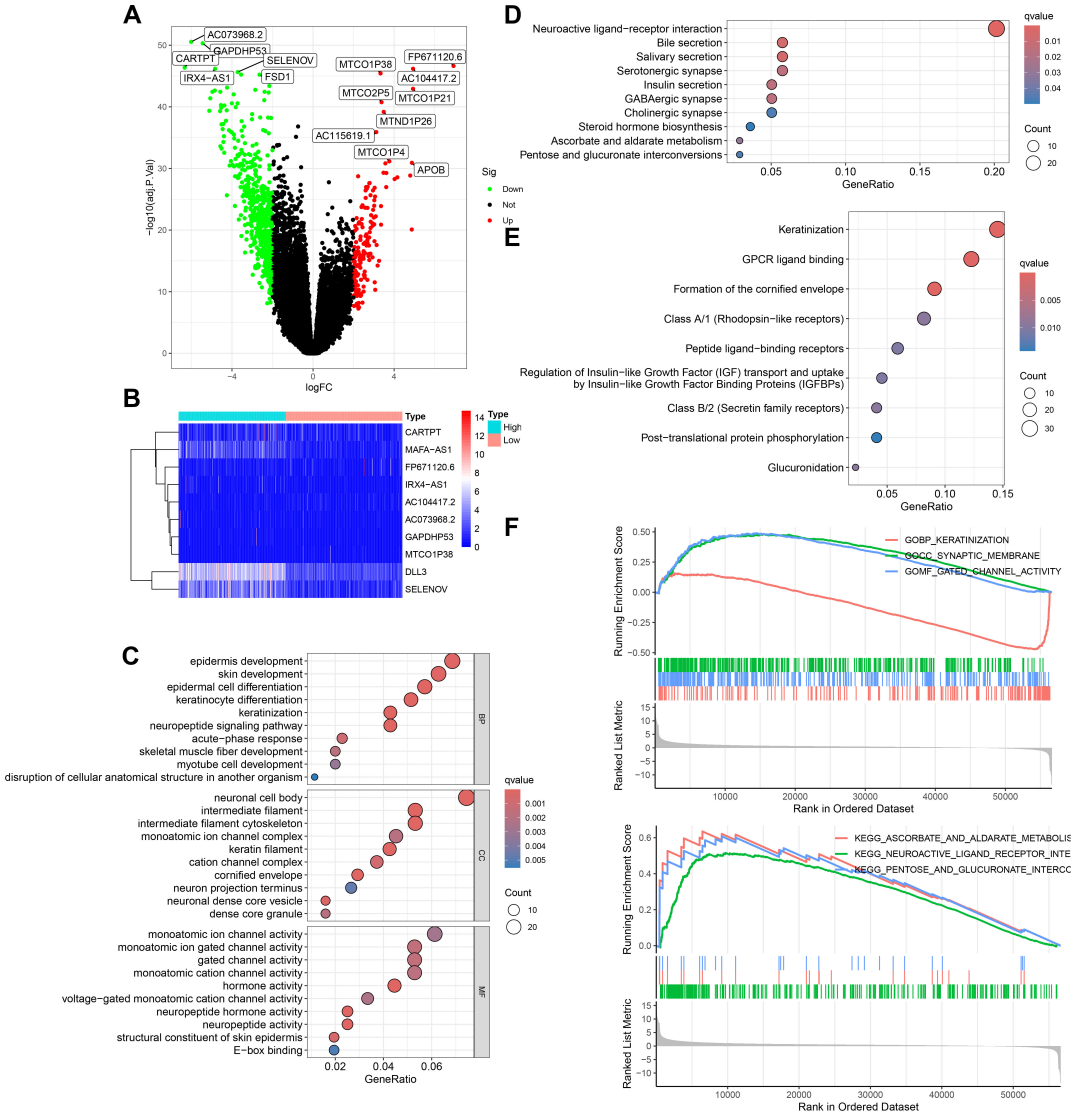
Transcriptomic and functional enrichment aanalysis uncovers the role of DLL3 in UCEC pathogenesis. **(A)** Differential gene expression volcano plot comparing high and low DLL3 expression groups in UCEC. Genes upregulated are highlighted in red, downregulated genes are shown in green. **(B)** Heatmap displaying the top 10 differentially expressed genes, showcasing the variance in gene expression between the high and low DLL3 expression groups (Including DLL3). **(C)** GO functional enrichment analysis illustrating the biological processes, cellular components, and molecular functions associated with the differentially expressed genes. **(D)** KEGG pathway enrichment analysis highlighting the significant pathways impacted by the differential gene expression. **(E)** Reactome pathway enrichment analysis revealing the involvement of differentially expressed genes in various biological pathways. **(F)** GSEA showing enriched gene sets in the high versus low DLL3 expression groups. The upper panel presents the enrichment of gene sets from the c5.go database (GO terms), and the lower panel displays the enrichment of gene sets from the c2.cp.kegg database (KEGG pathways).

To delve deeper into the transcriptomic characteristics and potential biological functions of DLL3 in UCEC, a bioinformatics analysis was conducted. The results, using TCGA data, divided the samples into high and low expression groups based on the median value of DLL3 expression. Differential gene analysis (LogFC>2) identified a total of 735 genes with significant changes, including 166 upregulated and 569 downregulated genes (**Figures 2A, B**). Furthermore, GO functional enrichment analysis revealed the top three enriched terms as epidermis development, neuronal cell body, and monoatomic ion channel activity (**Figure 2C**). KEGG enrichment analysis highlighted Salivary secretion, Bile secretion, and Neuroactive ligand−receptor interaction as the top three pathways (**Figure 2D**). Reactome enrichment analysis showed Formation of the cornified envelope, GPCR ligand binding, and Keratinization as the leading categories (**Figure 2E**). Additionally, GSEA c5.go functional enrichment pinpointed significant enrichment in GOBP KERATINIZATION, GOCC SYNAPTIC MEMBRANE, and GOMF GATED CHANNEL ACTIVITY. GSEA c2.cp.kegg functional enrichment revealed predominant enrichment in KEGG NEUROACTIVE LIGAND RECEPTOR INTERACTION, KEGG ASCORBATE AND ALDARATE METABOLISM, and KEGG PENTOSE AND GLUCURONATE INTERCONVERSIONS (**Figure 2F**). These results suggest that DLL3 may regulate cancer development and progression by influencing various biological functions.

### 3.3 Elevated expression of DLL3 in UCEC tissues and cell lines.

**Figure 3:**
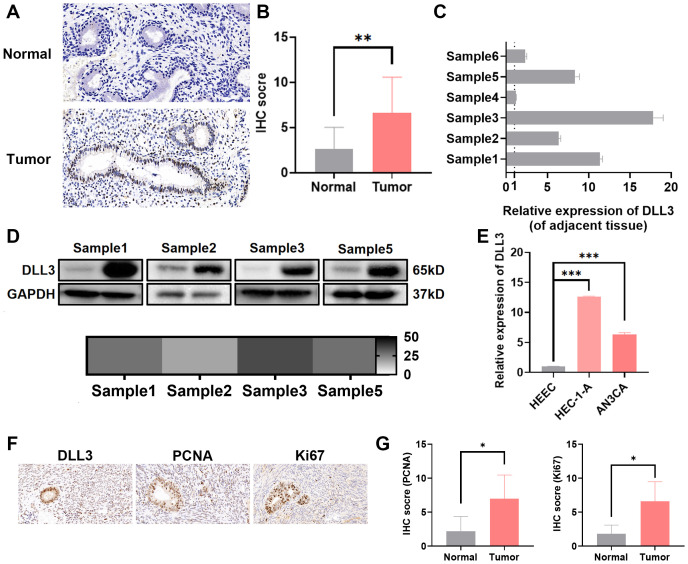
Elevated expression of DLL3 in UCEC tissues and cell lines. **(A)** Representative IHC images of DLL3 expression in tumor tissues and adjacent non-tumor tissues from six UCEC patients. n=6. **(B)** Quantification of IHC staining scores for DLL3 expression across six UCEC patients. n=6. **(C)** qRT-PCR analysis of DLL3 expression in tumor tissues and matched adjacent non-tumor tissues from the same six-patient cohort. n=6. **(D)** Western blot assay detecting DLL3 expression in tumor tissues and matched adjacent non-tumor tissues. The heat map shows the relative gray data from Western Blot experiments, with labels in monochrome from white to black, where black represents a fold change of 50. **(E)** The qRT-PCR assay detecting DLL3 expression in HEEC and UCEC cell lines. **(F)** Representative IHC images of proliferation markers PCNA and Ki67 in tumor tissues with high DLL3 expression from six UCEC patients. n=6. **(G)** Quantification of IHC staining scores for PCNA and Ki67 expression across the same six-patient cohort. n=6. The results presented were Mean ± SD. *p < 0.05, **p < 0.01, ***p < 0.001.

To confirm whether the expression level of DLL3 in UCEC aligns with database predictions, this study utilized immunohistochemistry, Western blotting, and other methods to assess DLL3 expression in collected samples from UCEC patients and corresponding adjacent non-tumor tissues. As indicated in **Figures 3A–D**, DLL3 expression was significantly higher in UCEC tissues compared to adjacent non-tumor tissues (**Figures 3A–D**), Besides, IHC results also showed higher PCNA and Ki67 expression in patients in the DLL3 high expression group or tumor group (**Figures 3F, G**). Further analysis through cell line studies, comparing HEEC with endometrial cancer cell lines (HEC-1-A and AN3CA), revealed that DLL3 expression levels in HEC-1-A and AN3CA cell lines were significantly elevated compared to HEEC (**Figure 3E**).

### 3.4 Knocking down DLL3 inhibits proliferation and migration abilities of UCEC *in vitro*

**Figure 4:**
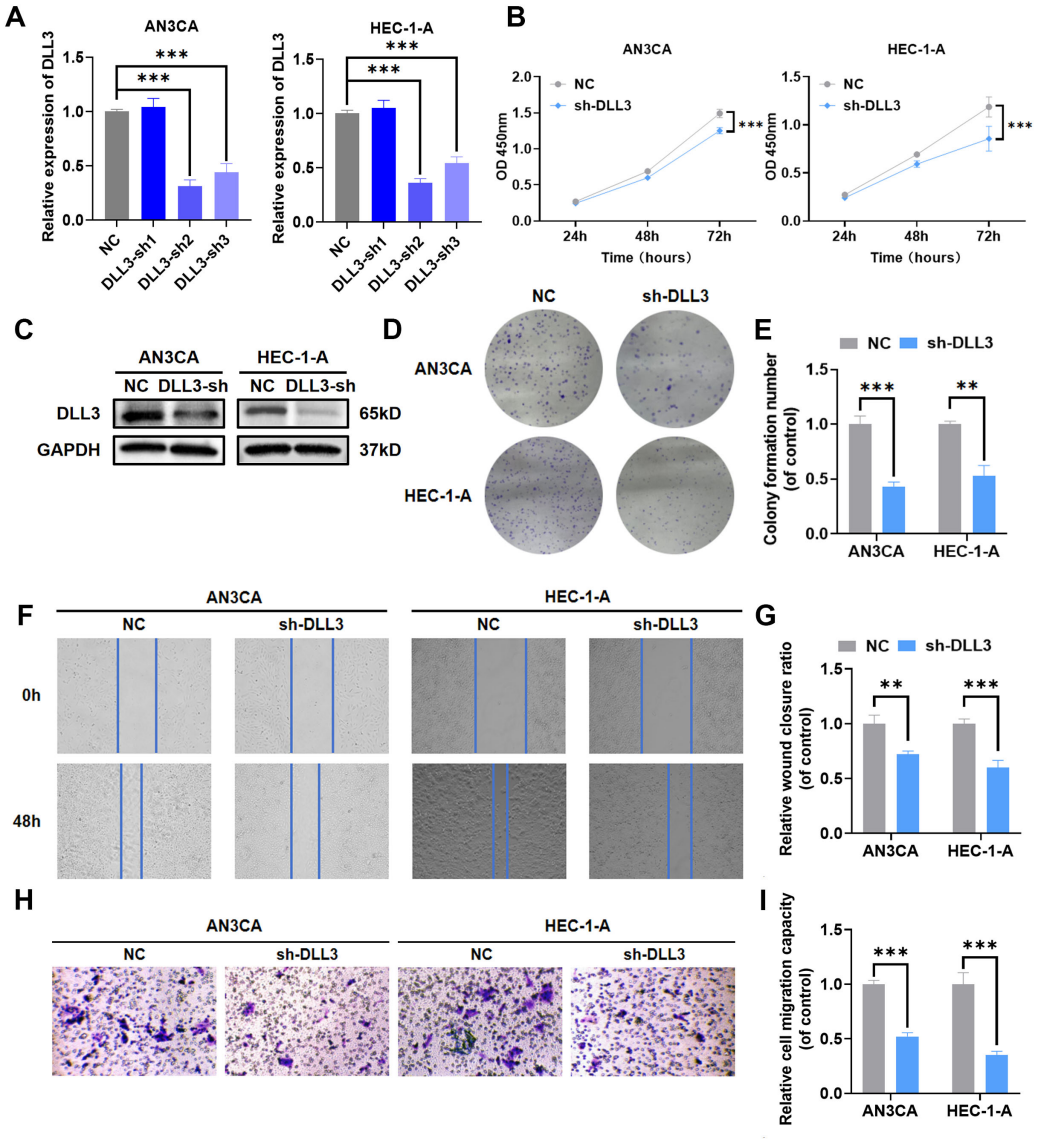
Knockdown of DLL3 impairs proliferation and migration capabilities in UCEC cell lines. **(A)** The qRT-PCR was used to detect the relative expression of DLL3. **(B)** Cell proliferation determined by the CCK-8 assay following DLL3 knockdown in cell lines. **(C)** The expression of DLL3 was detected by Western Blot. **(D)** Cell proliferation determined by the colony formation assay following DLL3 knockdown in cell lines. **(E)** Statistical graphs of the colony formation assay. **(F)** Cell migratory ability determined by the wound scratch assay following DLL3 knockdown in cell lines. **(G)** Statistical graphs of the wound scratch assay. **(H)** Cell migratory ability determined by the Transwell assay following DLL3 knockdown in cell lines. **(I)** Statistical graphs of the Transwell assay. The results presented were Mean ± SD. **p < 0.01, ***p < 0.001.

Previous functional enrichment results suggested that DLL3 may regulate the biological functions of UCEC by modulating the functionality of the intermediate filament cytoskeleton, which plays a crucial role in maintaining cell structure, crucial for the invasiveness and migration abilities of tumor cells. While previous studies have shown DLL3 regulates proliferation and migration in cancers such as gastric and prostate cancer, reports on its regulation in UCEC are absent. Therefore, DLL3 expression was knocked down in UCEC cell lines HEC-1-A and AN3CA. Knockdown efficiencies are presented in **Figures 4A, C** and **Supplementary Figure S1**. The results indicated that shRNA-2 had the highest knockdown efficiency (**Figure 4A**). And the western blot analysis further confirmed that shRNA-2 effectively reduced the expression of the DLL3 protein (**Figure 4C**, **Supplementary Figure S1**). Consequently, it was chosen for further experiments. Subsequently, the impact of DLL3 expression alteration on UCEC proliferative abilities was analyzed using CCK-8 and colony formation assays. Results indicated a significant reduction in UCEC cell proliferation following DLL3 knockdown (**Figures 4B, D, E**). Similarly, wound scratch and Transwell assays demonstrated reduced cell migration capabilities with decreased DLL3 expression (**Figures 4F–I**).

### 3.5 The suppression of DLL3 expression diminishes UCEC progression in a murine model

**Figure 5:**
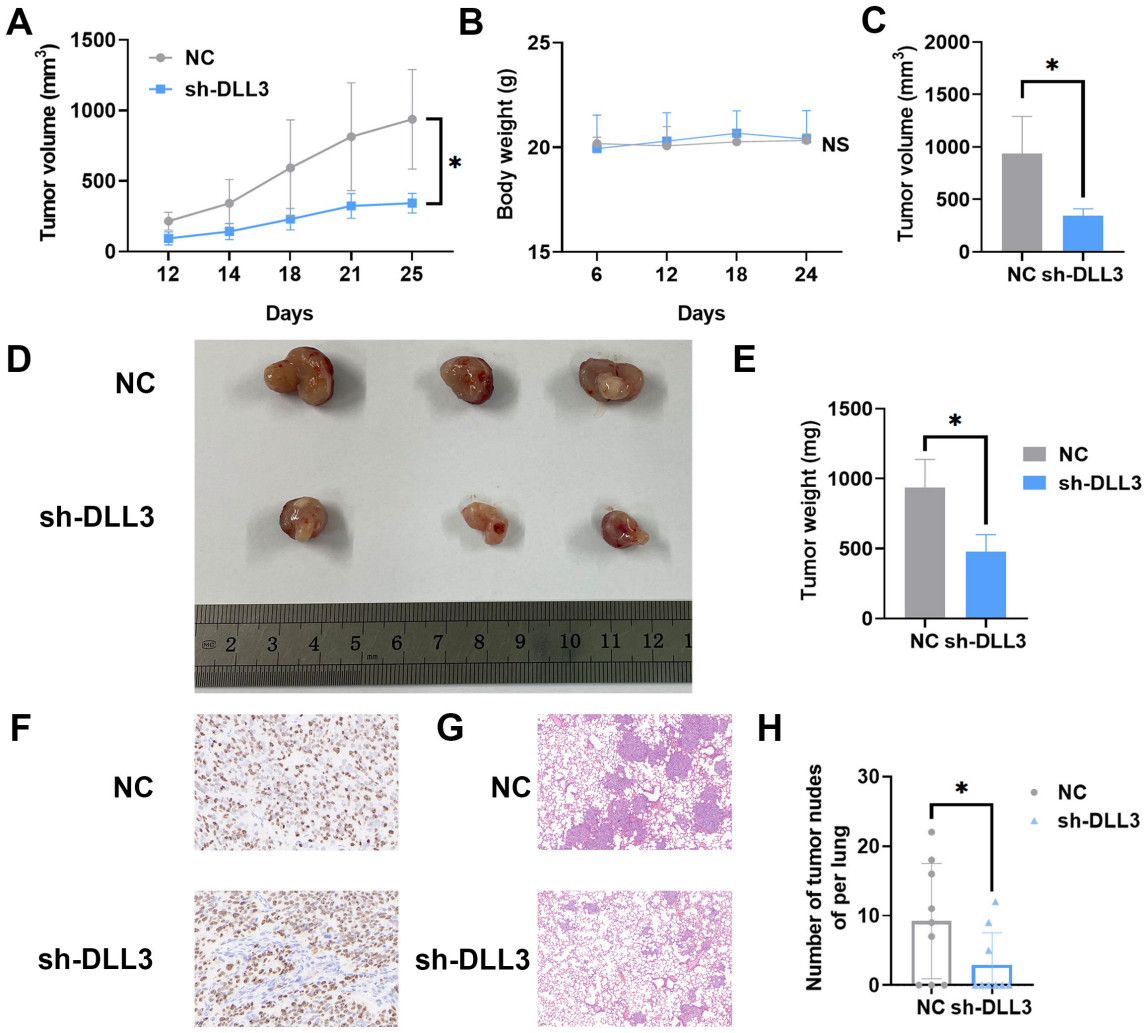
DLL3 Expression Suppression Reduces UCEC Progression in Nude Mice Models. **(A)** Tumor growth curves illustrating the progression of subcutaneous tumors over time n=3. **(B)** The curve of the body weight of nude mice n=3. **(C)** Tumor volume across different groups of subcutaneous tumor models. **(D)** Representative images of subcutaneous tumors harvested from each experimental group. **(E)** Tumor weight across different groups of subcutaneous tumor models. **(F)** Typical IHC for proliferation markers Ki67 in tumor sections. **(G)** Typical H&E staining of lungs. **(H)** Number of tumor nodules in the lungs. The results presented were Mean ± SD. *p < 0.05, NS > 0.05.

To explore the involvement of DLL3 in the *in vivo* progression of UCEC, nude mice were subcutaneously inoculated with HEC-1-A cells, wherein DLL3 was knocked down, along with a matched control group. Tumor volume was meticulously measured every six days, culminating in the euthanization of the mice 24 days after cell inoculation. Comparative analysis revealed a notable deceleration in the growth rate of tumors with DLL3 knockdown compared to controls, but the body weight exhibited no significant differences between them (**Figures 5A, B**). Subsequent examination of the excised tumors confirmed a significant reduction in both tumor weight and volume in the DLL3 knockdown group (**Figures 5C–E**). IHC analysis further underscored this finding, showing reduced proliferation markers Ki67 in the DLL3-suppressed tumors (**Figure 5F**), collectively indicating that DLL3 downregulation curtails tumor proliferation *in vivo*.

The HEC-1-A cells with diminished DLL3 expression were utilized alongside control group cells to evaluate their potential for metastasis *in vivo*. A pulmonary metastasis model was established by intravenously injecting tumor cells into nude mice. After 45 days post-injection, euthanasia was performed on the animals. Lung tissues were isolated and subjected to H&E staining. Subsequent examination under a microscope determined the presence of metastatic lesions in the lungs. As depicted in **Figures 5G, H**, within the control group, 6 out of 9 animals (66.6%) displayed pulmonary tumor nodules, whereas only 3 out of 9 animals (33.3%) injected with DLL3 suppressed HEC-1-A cells exhibited pulmonary tumor nodules. Furthermore, the nodules observed in animals from the sh-DLL3 group were notably smaller in size compared to those in animals inoculated with control group cells, indicative of diminished metastatic potential (**Figures 5G, H**). In summary, these findings suggest that inhibition of DLL3 expression attenuates the ability of HEC-1-A tumor cells to establish pulmonary metastases.

### 3.6 MiR-508-5p suppresses proliferation and migration in UCEC by targeting DLL3

**Figure 6:**
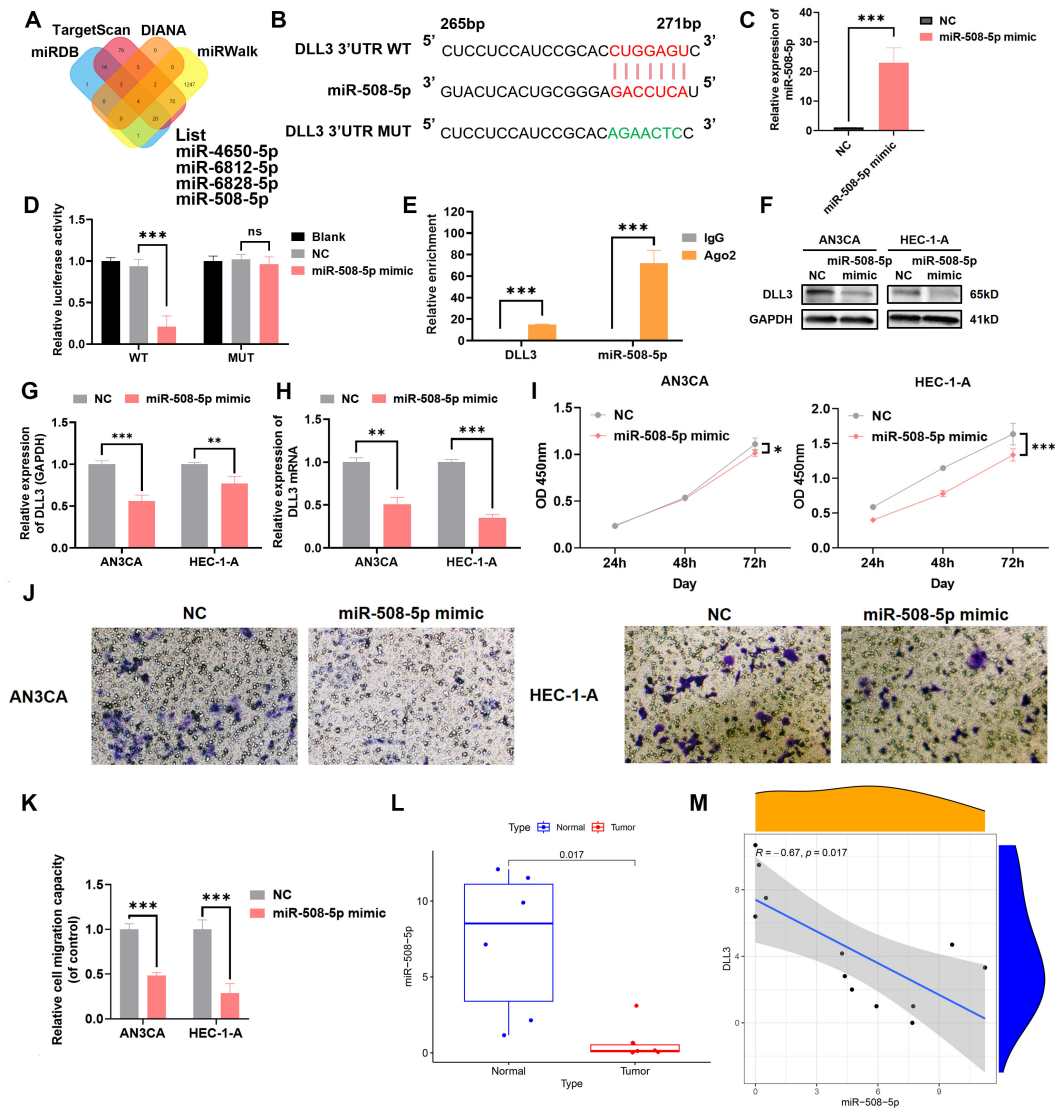
MiR-508-5p targets the 3’UTR of DLL3 mRNA and affects its expression. **(A)** Bioinformatics analysis identifying miRNAs with potential binding sites on DLL3. **(B)** Schematic representation of DLL3 mRNA highlighting the MiR-508-5p binding site. **(C)** The qRT-PCR was used to detect the relative overexpression of miR-508-5p. **(D)** Dual-luciferase reporter assay quantifying the impact of MiR-508-5p on luciferase activity linked to the DLL3 3’UTR. **(E)** RIP assay conducted with an anti-Ago2 antibody. **(F)** The expression of DLL3 was detected by Western Blot. **(G)** Western Blot analysis was quantified relative to GAPDH from three independent biological replicates. **(H)** The relative expression of DLL3 mRNA was detected by qRT-PCR. **(I)** Cell proliferation evaluated by CCK-8 assay in cells with altered MiR-508-5p expression. **(J)** Cell migratory ability detected by transwell migration assay. **(K)** Statistical graphs of the Transwell assay. **(L)** Analysis of MiR-508-5p expression across UCEC samples. n=6 **(M)**. Correlation analysis between miR-508-5p and DLL3 expression. The results presented were Mean ± SD. *p < 0.05, **p < 0.01, ***p < 0.001, NS > 0.05.

MiRNAs, endogenous RNA molecules approximately 22 nucleotides in length, play pivotal roles in gene expression regulation by targeting mRNAs. In the quest to identify miRNAs that regulate DLL3, bioinformatics analysis unveiled MiR-508-5p as a candidate with a potential binding site on the 3’ untranslated region (3’UTR) of DLL3 (**Figures 6A, B**). Subsequent validations using luciferase assays and RIP experiments established that DLL3 expression is modulated by MiR-508-5p targeting its 3’UTR (**Figures 6C–E**). Specifically, transfection of UCEC cell lines with MiR-508-5p mimic significantly attenuated the luciferase activity of the DLL3 wild type (WT), while the activity of a mutant DLL3 (MUT) lacking the binding site remained unchanged. Additionally, overexpression of MiR-508-5p led to a reduction in DLL3 expression within these cells (**Figures 6F–H**).

Empirical evidence has underscored that restoring MiR-508-5p expression notably hampers the proliferation and migration of UCEC cells (**Figures 6I–K**). Utilizing clinical UCEC samples, further investigation into the clinical significance of MiR-508-5p in UCEC revealed that the levels of MiR-508-5p were significantly reduced in UCEC tissues compared to adjacent non-tumor tissues (**Figure 6L**). And a clear negative correlation between MiR-508-5p and DLL3 expression was also revealed (**Figure 6M**). These findings reveal a potential mechanism by which MiR-508-5p inhibits DLL3 expression by targeting DLL3, thereby inhibiting the proliferation and migration tendency of UCEC cells.

**Figure 7:**
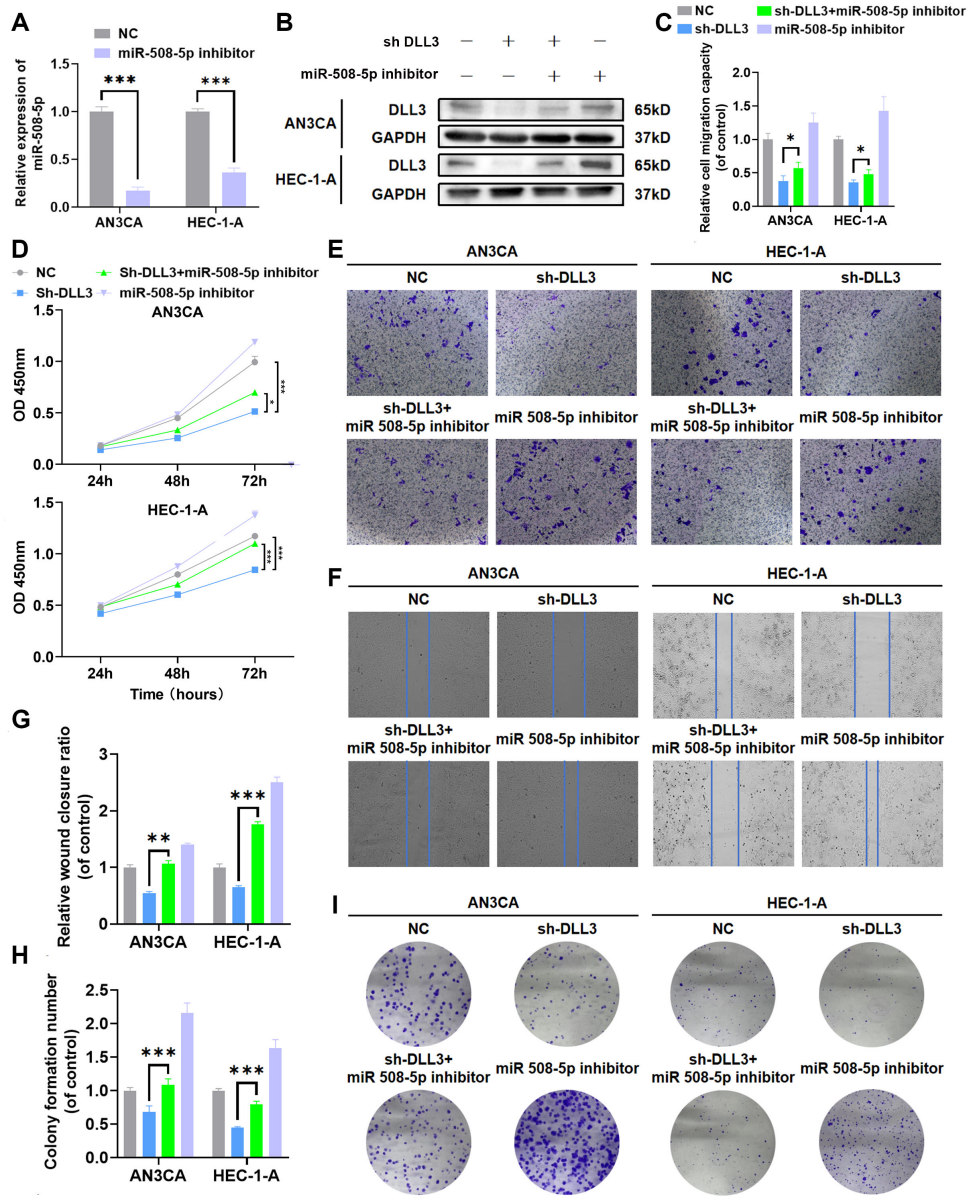
MiR-508-5p suppresses proliferation and migration in UCEC by targeting DLL3. **(A)** The qRT-PCR was used to detect the relative expression of miR508-5p. **(B)** The Western Blot was used to detect the expression of DLL3 protein. **(C)** Statistical graphs of the Transwell assay. **(D)** CCK-8 assay. **(E)** Transwell assay. **(F)** Wound scratch assay. **(G)** Statistical graphs of the wound scratch assay. **(H)** Statistical graphs of the colony formation assay. **(I)** Colony formation assay. The results presented were Mean ± SD. *p < 0.05, **p < 0.01, ***p < 0.001.

To further investigate whether the deletion of MiR-508-5p leads to increased expression of DLL3, thereby promoting the proliferation and migration of UCEC cells, we conducted rescue experiments. Western blotting results revealed that, contrary to the previous findings with the introduction of MiR-508-5p mimic into UCEC cells, the addition of MiR-508-5p inhibitor significantly increased the expression levels of DLL3 (**Figures 7A, B**, **Supplementary Figure S2**). Concurrently, CCK-8, wound scratch, colony formation and Transwell assays further demonstrated a significant increase in the proliferation and migration abilities of UCEC tumor cells exposed to the MiR-508-5p inhibitor compared to the negative control group (**Figures 7C–I**). However, this enhanced capability was partially reduced upon blockade of DLL3 (**Figures 7C–I**). These results collectively suggest that MiR-508-5p inhibits DLL3 expression by directly targeting DLL3, consequently suppressing the proliferation and migration of UCEC cells.

### 3.7 Construction of engineered MiR-508-5p overexpressing exosomes and delivery of miRNA to tumor tissue

**Figure 8:**
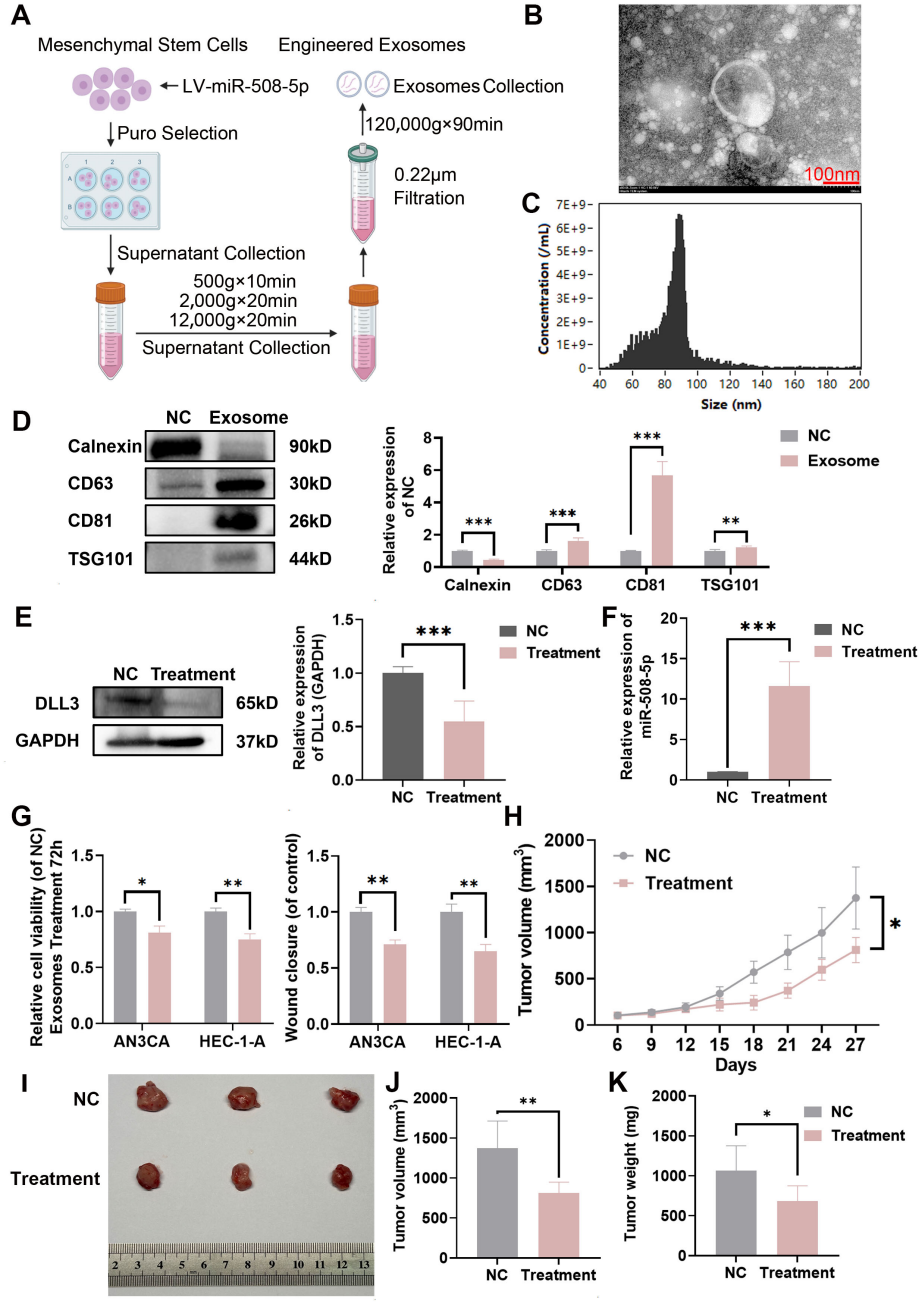
Construction of engineered miR-508-5p overexpressing exosomes and delivery of miRNA to tumor tissue. **(A)** Preparation and isolation methods of engineered miR-508-5p overexpressing exosomes. **(B)** Representative TEM images of engineered miR-508-5p overexpressing exosomes. **(C)** FCM analysis of particle size and concentration of engineered miR-508-5p overexpressing exosomes. **(D)** Western blot analysis detecting the expression of exosome markers CD63, CD81, TSG101 and Calnexin, quantified relative to NC from three independent biological replicates. **(E)** Western blot analysis of DLL3 expression following engineered exosomes treatment, quantified relative to GAPDH from three independent biological replicates. **(F)** qRT-PCR analysis of the relative expression of miR-508-5p following treatment with engineered exosomes. **(G)** CCK-8 assay and wound scratch assay following treatment with engineered exosomes. **(H)** Tumor growth curves illustrating the progression of subcutaneous tumors over time. Treatment was initiated when the tumors reached 100 mm^3^. Injections were administered intratumorally, three times in total, once every other day, at a dose of 10 μg per mouse (n=3). **(I)** Representative images of subcutaneous tumors harvested from each experimental group. **(J)** Tumor volume across different groups of subcutaneous tumor models. **(K)** Tumor weight across different groups of subcutaneous tumor models. The results presented were Mean ± SD. *p < 0.05, **p < 0.01, ***p < 0.001.

To efficiently deliver miRNA to tumor tissues, we developed engineered exosomes overexpressing MiR-508-5p based on MSCs. The specific isolation method is illustrated in **Figure 8A**. The extracted exosomes were observed using transmission electron microscopy, which revealed the characteristic bilayer membrane of exosomes (**Figure 8B**). Nano-flow cytometry showed that the particle size of the prepared exosomes ranged from 60 to 120nm, with a mean size of 84.6 (**Figure 8C**). Western blotting confirmed that exosome markers were higher in exosomes compared to donor cells (**Figure 8D**). Furthermore, after 4 hours of co-culture, MiR-508-5p expression in UCEC cells was significantly increased compared to the PBS control group (**Figure 8F**), indicating that the engineered exosomes effectively and precisely delivered miRNA to the tumor cells and restored MiR-508-5p expression. After 48 hours of co-culture, DLL3 expression was also significantly reduced (**Figure 8E**). Functional assays demonstrated that treatment with engineered MiR-508-5p overexpressing exosomes significantly decreased UCEC cell proliferation and migration (**Figure 8G**).

Additionally, to better evaluate the therapeutic efficacy of the engineered exosomes *in vivo*, a subcutaneous xenograft model of HEC-1-A was established. Once the tumors reached a volume of 100 mm³, tumor-bearing mice were treated by intratumoral injection with 10 μg of exosomes per mouse, administered every other day for a total of five injections. The endpoint for euthanasia was set at a tumor volume of 1500 mm³. Results showed that engineered MiR-508-5p overexpressing exosomes significantly reduced tumor burden in mice (**Figures 8H–K**).

## 4 Discussion

Known for its regulatory function in somatic and neurogenic development (28), DLL3 is an inhibitory ligand within the Notch signaling pathway (29, 30). In small cell lung cancer (SCLC), altered DLL3 expression has a notable impact on patient survival outcomes (31). Preclinical investigations and phase I trials have indicated that antibody drugs targeting DLL3 hold promise for SCLC treatment (31). Nevertheless, research also suggests that DLL3 can exert tumor-suppressive effects, as observed in studies of hepatocellular carcinoma and glioblastoma (32–35). This demonstrates the capacity of DLL3 to function as either an oncogene or tumor suppressor, a role that is dictated by the specific tumor microenvironment and cellular context. Thus, understanding the role of DLL3 in various cancers is crucial. In this study, bioinformatics analysis revealed significant differences in DLL3 expression across tissues. Using qRT-PCR, Western blotting, and IHC, we observed a notable increase in DLL3 expression in UCEC tissues compared to adjacent non-tumor tissues. Furthermore, retrospective prognostic data from UCEC patients indicated that high levels of DLL3 correlate with poor prognosis. These findings suggest that DLL3 may act as an oncogene in UCEC cells. In UCEC cell lines, DLL3 expression was knocked down, resulting in a significant decrease in the proliferative and migratory capabilities of the tumor cells, consistent with the bioinformatics predictions. These experimental results align with those of Furuta M, who found that knocking down DLL3 in SCLC reduced cell migration and invasion, whereas overexpression increased these activities (36). Similarly, Ding X found that DLL3 was upregulated in A2058 cells stimulated with LPS or TNF-α, and knocking down DLL3 inhibited LPS-induced inflammation, migration, and invasion in these cells, along with a decrease in Matrix Metalloproteinase 1 (MMP1), Matrix Metalloproteinase 9 (MMP9), and Vascular Endothelial Growth Factor (VEGF) (37).

Like transcription factors, non-coding RNAs can also regulate gene expression. MiRNAs, small non-coding RNAs discovered in 1993, have been shown to modulate the activity of genes (12, 38). Although miRNAs are encoded by only about 3% of human genes, they can regulate approximately 30% of human protein-coding genes (39). In the classical biosynthetic pathway, miRNAs regulate gene expression by binding to mRNA through Watson-Crick pairing (40), influencing various biological functions, including proliferation, differentiation, and apoptosis (11, 41, 42). In this study, changes in the expression of DLL3 were accompanied by significant alterations in MiR-508-5p levels in UCEC samples. Analysis using multiple bioinformatics miRNA prediction databases identified potential MiR-508-5p binding sites on the 3’UTR of DLL3. Based on this evidence, we hypothesized that DLL3 might be a potential target gene of MiR-508-5p in UCEC. Dual-luciferase assays revealed that the relative luciferase activity in the MiR-508-5p mimic + DLL3 3′-UTR group was significantly reduced compared to the control group, while the activity in the mutation group remained unchanged. These results confirm that DLL3 is a target gene of MiR-508-5p in UCEC. Restoring the expression of MiR-508-5p in UCEC cells led to downregulation of DLL3 levels, whereas silencing MiR-508-5p had the opposite effect. Further validation was conducted through rescue experiments, which confirmed that silencing MiR-508-5p promoted the proliferation and migration of UCEC cells by targeting the DLL3 gene. This is consistent with previous studies indicating that miR-508 can regulate cancer progression, such as in lung adenocarcinoma, cholesteatoma, and esophageal squamous cell carcinoma, where its expression is typically significantly reduced (43–45). It is worth noting that a previous study by Chen et al. demonstrated that miR-508-5p functions as a tumor suppressor in endometrial cancer stem cells (ECSC) using ECC and ECSC cell lines. Our findings are consistent with Chen’s results, reinforcing the role of miR-508-5p as a critical tumor suppressive factor in UCEC (46). However, while Chen utilized ECC and ECSC models, our study employed the HEC-1-A and AN3CA cell lines. This difference in cell line selection may lead to variations in cellular responses and molecular mechanisms, providing a broader understanding of miR-508-5p’s efficacy across different endometrial cancer models. Together, these studies highlight the potential of miR-508-5p as a universal therapeutic target for the treatment of endometrial cancer.

Compared to other gene therapies or biological treatments, exosomes serve as non-toxic “natural” delivery vehicles capable of transferring therapeutic molecules (such as nucleic acids and recombinant proteins) into cancer cells. Increasing research shows that exosomes can carry “cargo” to target cells for therapeutic purposes. For instance, therapeutic miRNA or miRNA expression vectors can be transfected into parental cells, allowing the miRNA to be endogenously encapsulated in exosomes. The secreted exosomes can then be isolated to resensitize colon cancer to chemotherapeutic drugs (47). Exosomes derived from ADMSCs can impair cisplatin (DDP) resistance in both parental and DDP-resistant breast cancer cell lines. Mechanistic studies indicate that ADMSC-derived exosomes enhance DDP sensitivity by downregulating Solute Carrier Family 9 Member A1 (SLC9A1) through miR-1236 (48). In this study, we employed a similar strategy to construct therapeutic exosomes and preliminarily validated their tumor-suppressive effects both *in vitro* and *in vivo*. In UCEC cells treated with these exosomes, a significant reduction in cell proliferation and migration was observed. Additionally, the tumor burden in UCEC-bearing mice was notably decreased.

This study has its limitations, as is well-known that existing research models have significant flaws. Rodents do not menstruate and therefore cannot accurately replicate human conditions. Although primates do experience spontaneous menstruation, they are costly to maintain (49, 50). Moreover, nude mice lack T lymphocytes due to abnormal thymus development, leading to a partially deficient immune system, which may not fully reflect the true *in vivo* expression of DLL3 (51). Consequently, this study employed only subcutaneous xenograft models. While knocking down DLL3 in HEC-1-A reduced tumor burden, the results remain constrained by these limitations. Our future research will further explore the molecular mechanisms of DLL3 expression in regulating UCEC using organ-like models of human endometrium and existing humanized immune system mouse models based on immunodeficient mice. Furthermore, since a single miRNA can target hundreds of mRNAs, thereby regulating an entire protein network (52), and conversely, one mRNA can be regulated by several miRNAs, there are also other endogenous competitive RNAs (ceRNAs) such as Circular RNAs (circRNAs) and Long Non-coding RNAs (lncRNAs)that may act as “sponges” and interfere with specific miRNA-mRNA interactions (53). Therefore, while *in vitro* results show that MiR-508-5p can affect proliferation and migration in UCEC cells by influencing DLL3 expression, whether MiR-508-5p is the primary factor impacting DLL3 remains to be further confirmed. Additionally, although we developed engineered exosomes overexpressing MiR-508-5p based on MSCs, there is still a considerable distance to clinical application. The first issue is production yield. In most studies, the yield of exosomes from 1 mL of culture medium is usually less than 1 μg of exosomal protein (54), while the effective dose of exosomal protein is typically 10-100 μg per mouse (55). Secondly, the use of exosomes in clinical trials must comply with GMP standards, and different purification methods often result in variations in their physicochemical properties (56). Thirdly, there are potential risks associated with exosome therapy. Since the biological effects of exosomes are mediated through uptake by target cells, it is essential to clarify and control the biosafety of host cell-derived exosomes (57).

In summary, the results demonstrate that DLL3 is a potential target gene of MiR-508-5p in UCEC, and there is a negative correlation between MiR-508-5p and DLL3 in UCEC. Restoring MiR-508-5p in UCEC cell lines by targeting DLL3 reduces cell proliferation and migration, thereby slowing the progression of UCEC. Additionally, we developed a therapeutic strategy based on MSCs engineered to overexpress MiR-508-5p. This approach restores MiR-508-5p expression in UCEC cells, reduces DLL3 expression, and consequently diminishes cell proliferation and migration. This study provides new insights into the function of the MiR-508-5p/DLL3 axis in the development of UCEC, and targeting its expression could offer novel perspectives for the prevention and treatment of UCEC.

## Data Availability

The original contributions presented in the study are included in the article/**Supplementary Material**. Further inquiries can be directed to the corresponding author/s.
