# Supplementary material for: Engineered exosomes restore miR-508-5p expression in uterine corpus endometrial carcinoma and reduce tumor progression and metastasis by targeting DLL3
Source: Front Oncol. 2025 Feb 24;15:1532564. doi: 10.3389/fonc.2025.1532564 (PMC11891050; doi:10.3389/fonc.2025.1532564)
Supplement: Supplementary file 1 [file DataSheet1.docx]

**Engineered Exosomes Restore miR-508-5p Expression in Uterine Corpus Endometrial Carcinoma and Reduce Tumor Progression and Metastasis by Targeting DLL3**

Yue-Ying Li^1, 2^, Hui Liu^3^, Jia-Lu Feng^4^, Wen-Yan Tian^1, 2*^, Juan Du^5*^, Li-Ping Zhang^5*^

*Corresponding author:

Wen-Yan Tian, Department of gynecology, Department of Gynecology and Obstetrics, Tianjin Medical University General Hospital, Tianjin, China, Tianjin Key Laboratory of Female Reproductive Health and Eugenic, Tianjin Medical University General Hospital, Tianjin, China. Email: tianwenyan@tmu.edu.cn.

Juan Du, Department of gynecology, Wuhan Children’s Hospital (Wuhan Maternal and Child Healthcare Hospital), Tongji Medical College, Huazhong University of Science & Technology, Hubei, China. Email: dujuan19109@163.com.

Li-Ping Zhang, Department of gynecology, Wuhan Children’s Hospital (Wuhan Maternal and Child Healthcare Hospital), Tongji Medical College, Huazhong University of Science & Technology, Hubei, China. Email: syzlp518@163.com

**Supplementary Figure 1**

**
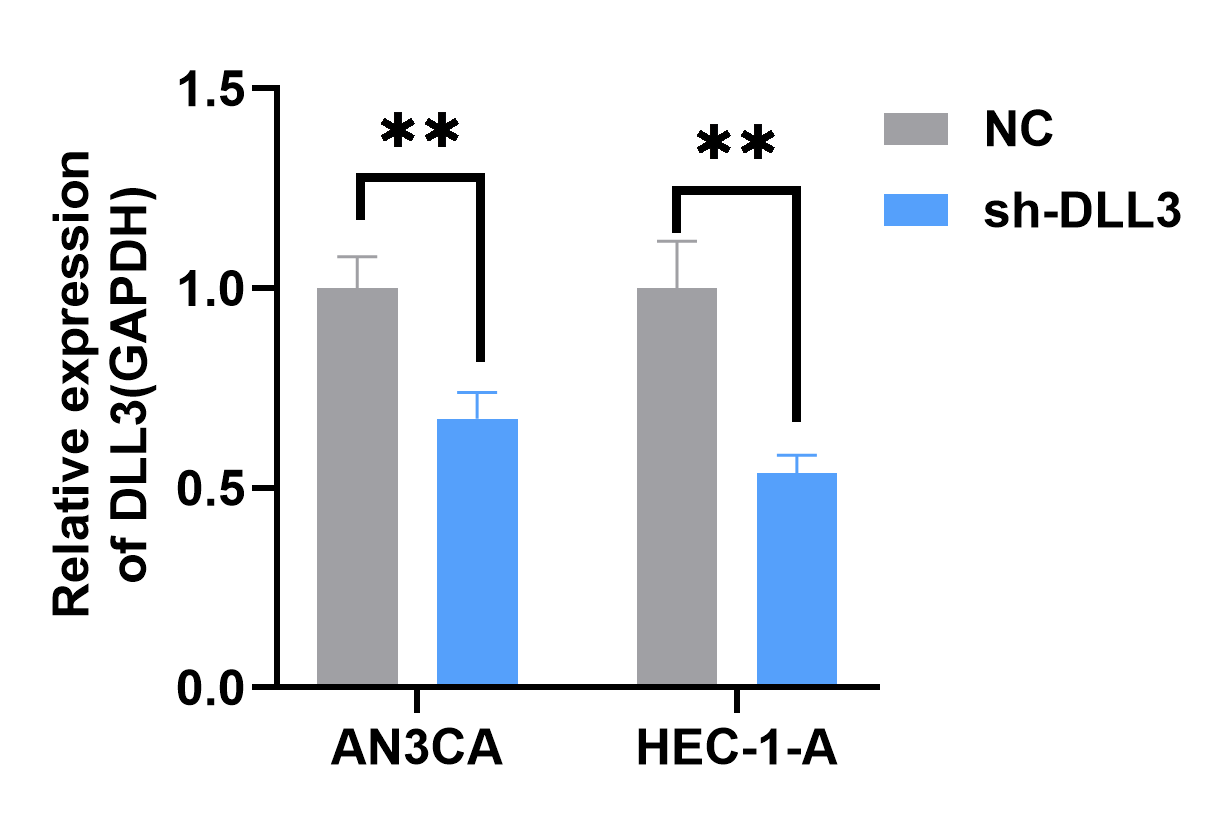
**

Supplementary Figure 1. Western Blot analysis (Figure 4C) was quantified relative to GAPDH from three independent biological replicates.

**Supplementary Figure 2**


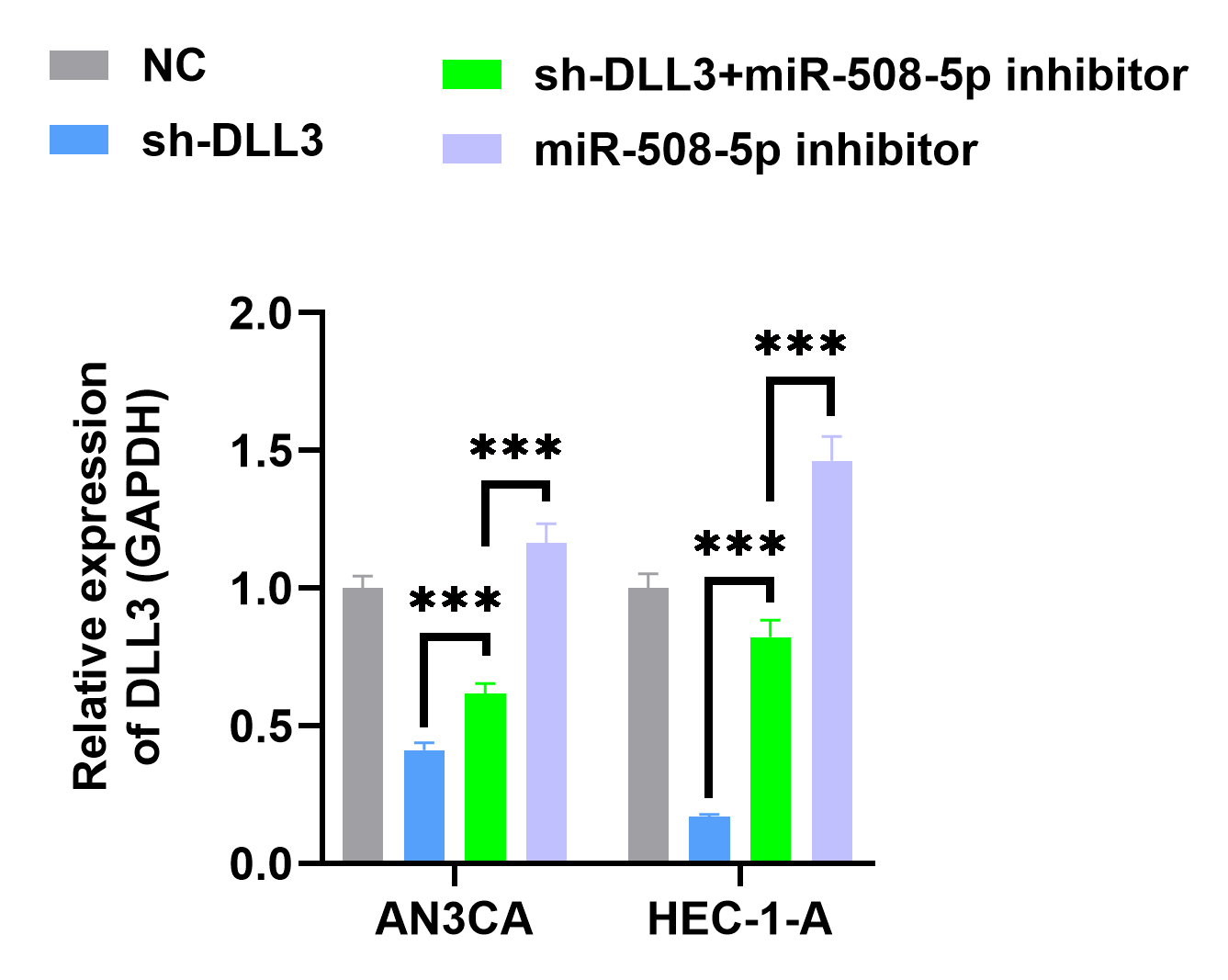


Supplementary Figure 2. Western Blot analysis (Figure 7B) was quantified relative to GAPDH from three independent biological replicates.
